# Why Synchrony Matters during Mother-Child Interactions: A Systematic Review

**DOI:** 10.1371/journal.pone.0113571

**Published:** 2014-12-03

**Authors:** Chloë Leclère, Sylvie Viaux, Marie Avril, Catherine Achard, Mohamed Chetouani, Sylvain Missonnier, David Cohen

**Affiliations:** 1 Institut des Systèmes Intelligents et de Robotiques, CNRS, UMR 7222, Université Pierre et Marie Curie, Paris, France; 2 Département de Psychiatrie de l'Enfant et de l'Adolescent, AP-HP, Hôpital Pitié-Salpêtrière, Paris, France; 3 Université Paris V René Descartes, Département de Psychopathologie, Boulogne, France; Harvard Medical School, United States of America

## Abstract

**Background:**

Assessment of mother-child interactions is a core issue of early child development and psychopathology. This paper focuses on the concept of “synchrony” and examines (1) how synchrony in mother-child interaction is defined and operationalized; (2) the contribution that the concept of synchrony has brought to understanding the nature of mother-child interactions.

**Method:**

Between 1977 and 2013, we searched several databases using the following key-words: « *synchrony* » « *interaction* » and « *mother-child* ». We focused on studies examining parent-child interactions among children aged 2 months to 5 years. From the 63 relevant studies, we extracted study description variables (authors, year, design, number of subjects, age); assessment conditions and modalities; and main findings.

**Results:**

The most common terms referring to synchrony were mutuality, reciprocity, rhythmicity, harmonious interaction, turn-taking and shared affect; all terms were used to characterize the mother-child dyad. As a consequence, we propose defining synchrony as a dynamic and reciprocal adaptation of the temporal structure of behaviors and shared affect between interactive partners. Three main types of assessment methods for studying synchrony emerged: (1) global interaction scales with dyadic items; (2) specific synchrony scales; and (3) micro-coded time-series analyses. It appears that synchrony should be regarded as a social signal *per se* as it has been shown to be valid in both normal and pathological populations. Better mother-child synchrony is associated with familiarity (vs. unknown partner), a healthy mother (vs. pathological mother), typical development (vs. psychopathological development), and a more positive child outcomes.

**Discussion:**

Synchrony is a key feature of mother-infant interactions. Adopting an objective approach in studying synchrony is not a simple task given available assessment tools and due to its temporality and multimodal expression. We propose an integrative approach combining clinical observation and engineering techniques to improve the quality of synchrony analysis.

## Introduction

### Early infant-caregiver interactions

Since Itard's description of the wild child [1], parent-child interactions and the social environment have been widely acknowledged as playing a central role in early developmental processes [2]. Aside from serving as a response to basic infant needs (e.g., feeding), the quality of parent-child relationship has been implicated in children's social, emotional and cognitive development for years [3], [4]. Studies have shown significant correlations between the quality of the parent-child relationship and children's developmental outcomes (e.g., social competence [5], [6] and emotion regulation [7]–[9]). As a consequence, dysregulation in parent-child interactions has been implicated in the development of children's problematic behaviors [9], [10]. Additionally, atypical parent-child interactions are suspected to provide initial evidence of pervasive developmental impairments, such as autism, among infants [11]–[14].

Aside from individual behaviors and characteristics, understanding parent-child interactions is at the heart of early childhood psychopathology. Perinatal clinicians and researchers have conducted experiments and developed theories about early parent-child interactions. Initial studies focused primarily on mother-infant interactions, however the role of father-child (or other caregiver-child) interactions is now widely accepted. Interactions between infants and their partners occur at three different levels: behavioral, affective, and fantasy [15]. The behavioral level is the level most often studied due to its experimental accessibility, however it is not a simple task to describe parent-child behavioral interactions because there are multiple modalities of interaction to explore and classify. First, the interactive partnership between an infant and caregiver (usually called a “dyad”) has to be defined and explored as a single unit. Second, given that the relationship between an infant and his caregiver is bidirectional in nature, the dyad should be thought of as a dynamically interacting system [16]. An infant can influence the care he receives from the caregiver by the ways he behaves [17], [18]. Third, given the dynamic relationship between an infant and his caregiver, a specific interest in the flow characterizing the exchange of information during infant-caregiver interactions has emerged [19], [20], leading to the study of rhythm (*meaning balance between partners*) [21]–[23], reciprocity (*meaning partners' ability to show adaptation to each other*) [24], [25], and synchrony (*meaning the dynamic and reciprocal adaptation of the temporal structure of behaviors between interactive partners*) [26]. The recent discovery of both biological correlates of behaviorally synchronic phenomena [27] and statistical learning [28], [29] has validated the crucial value of studying synchrony during child development [2], [26].

### Synchrony

Synchrony is an important concept relevant to diverse domains in physical, biological and social science. The construct of synchrony has been applied to a range of phenomena, from the micro-level of cells, neurons, and genes [30], [31] and intermediate-level of interactive partners' brains [27], to the macro-level of population growth and weather change [32] in addition to the mental realm [33]. In the field of mother-child interactions, the dynamic and reciprocal adaptation of the temporal structure of behaviors between interactive partners defining synchrony implies the following [34]: (i) behaviors include verbal and non-verbal communicative and emotional behaviors (e.g., gestures, postures, facial displays, vocalizations, and gazes). (ii) Synchronous interactions entail coordination between partners and intermodality. Caregivers and their children are able to respond to each other using different modalities starting from birth [35], [36]. Thus, synchrony differs from mirroring or the chameleon effect. Instead, synchrony describes the intricate ‘dance’ that occurs during short, intense, playful interactions; it builds on familiarity with the partner's behavioral repertoire and interaction rhythms; and it depicts the underlying temporal structure of highly aroused moments of interpersonal exchange that are clearly separated from the stream of daily life [23], [25], [37]–[39].

Despite the similarities between synchrony and other established constructs in the mother–child relationship, synchrony is different in a number of meaningful ways. Synchrony encompasses both the mother's and the child's responsivity and their emotional capacity to respond each other. During early development, synchrony involves a matching of behavior, emotional states, and biological rhythms between parents and infants that together forms a single relational unit (dyad) [26]. Affiliative bonds, defined as selective and enduring attachments, are formed on the basis of multiple genetic, hormonal, brain, autonomic, epigenetic, behavioural, and mental processes that coordinate to establish the parent–infant bond [40]. Oxytocin, considered to be the bonding hormone, appears to enhance physiological and behavioral readiness for social engagement in parent-infant interactions [19]. Its biology is not fully elucidated but is, in part, related to epigenetic mechanisms. Oxytocin (OT) is synthesized in the paraventricular and supraoptic nuclei of the hypothalamus. OT is released into both the peripheral circulation and the extracellular space, resulting not only in local action but also in diffusion through the brain to reach distant targets. OT receptors are localized in different areas including the amygdala, hippocampus, striatum, supra-chiasmatic nucleus, and brainstem. The fact that OT has peripheral and central functions does not imply that the central and peripheral release are necessarily associated [41].

Understanding the dynamics of mother-infant interactions and identifying synchronic patterns within mother-child dyads are important to promoting healthy relationships [42]. In typically developing children, the quality of social interactions depends on an active dialogue between the parent and the infant and is based on the infant's desire to be social and the parent's capacity to be attuned [43], [44]. Synchrony can therefore be defined as the temporal coordination of micro-level relational behaviors into patterned configurations that become internalized and serve to shape infant development over time and repeated experience [45]. Bernieri [46] proposed classifying definitions of synchrony that involve some notion of behavioral adjustment or entrainment to one another, into three categories. The first category is based on biological rhythms and defines synchrony as the degree of congruence between infant-caregiver behavioral cycles. The second category operationalizes synchrony as the quantity of simultaneous behaviors. The third category defines synchrony as a perceptual social phenomenon where the essential feature is the apparent unification of behavioral elements into a meaningful described “whole” (i.e., a synchronous event as a perceptual unit).

Originally conceptualized and studied by developmental psychologists, the concept of synchrony is now relevant to many different fields of study including social signal processing, robotics and machine learning [47], [48]. According to its conceptual framework, synchrony can be defined in many ways. However, Delaherche et al. [34] recently proposed that, in most cases, one should distinguish between *what* is assessed (i.e., modalities such as body movement, gaze, smile, and emotion) and *how* the temporal link between partners' different modalities of interaction are assessed (i.e., speed, simultaneity, smoothness). Therefore, synchrony has been measured in many different ways due to its broad range of theoretical applicability and has been applied to the study of parent-child interactions among both typically developing infants and clinical populations. In this study, we systematically review how the concept of synchrony has been defined in the study of early human interactions, limiting our review to studies involving infants and toddlers aged two months to 5 years and mothers, and what the associated main findings and contributions have been for understanding early child development.

## Methods

### Searching and selection of studies

**Figure 1:**
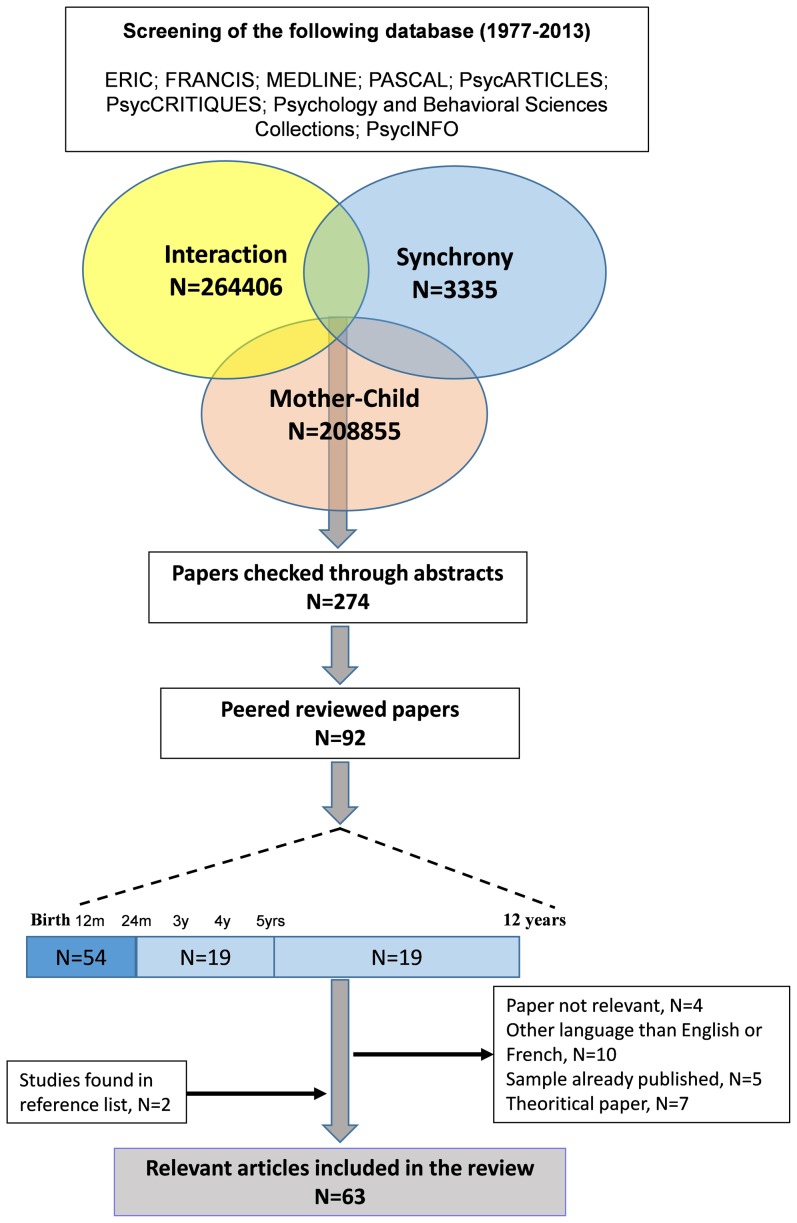
Diagram flow of the study.

An electronic search was undertaken, covering the following databases: ERIC; FRANCIS; MEDLINE; PASCAL; PsycARTICLES; PsycCRITIQUES; PsycEXTRA; Psychology and Behavioral Sciences Collections; and PsycINFO. This ensured that a range of psychology references with multiple theoretical background were included. We searched the literature for research articles published between 1977 and 2013 using the following key-words: « synchrony », « mother-child » and « interaction ». All articles were peer-reviewed. We examined the mother-child dyad because this dyad type has been the most thoroughly examined with respect to synchrony. A diagram summarizing the literature search process is provided in Figure 1. We used the following criteria: (1) studies investigating synchrony during mother child interaction; (2) studies using a specific tool for quantification of synchrony; (3) studies including children aged between 2 months and 5 years of age. (4) Finally, we excluded single case reports. Out of the 92 articles found through our initial search using criteria 1, 2 and 4, we selected 61 studies which included children aged between 2 months and 5 years of age. This age window was selected based on the following: (1) this age group represents a significant developmental period of communicative abilities with care-giver; (2) children greater than 2 months of age possess a greater capacity to respond with multiple modalities; and (3) this age group is awake for longer periods of time. We added 2 studies which were found by checking the reference lists of the selected studies. Several studies with mixed age samples (those including both children within our age inclusion criteria as well as infants younger than 2 months and/or children over 5 years of age) were excluded from our study. We also excluded 4 studies because synchrony did not appear relevant to the studies (e.g. not focused on synchrony, or theoretical) [45], [49]–[51]. Of note, we did not find other reviews sharing our goals.

### Data extraction

A three-step process was undertaken to review the studies evaluating synchrony in caregiver-child interactions. All information was gathered in an electronic database. Two of the study co-authors (CL and SV) blindly extracted the study information. Disagreement between raters was examined, and final extraction was validated through consensus with a third co-author (DC, CA or MC). Each of the two study co-authors (CL and SV) first provided a general description of the articles and systematically extracted the following data: authors and year; study design (e.g., prospective; selected population); number of subjects/dyads; mothers' characteristics (age, socio-economic status, parity and ethnicity); children's mean age; assessment modalities (e.g., behavioral annotation); and main findings including those regarding synchrony. Second, we examined how synchrony was characterized in terms of definitions and terms used in each of the articles. This was done to better capture how synchrony was conceptualized by authors according to their theoretical background. Third, we systematically detailed how synchrony was assessed, differentiating between both the method of annotation used (e.g., specific grid) and computation (e.g., time-series analysis). Other information was extracted from each article to provide detail on assessment conditions: setting of interactions (place, duration, order, video recording) and measurement components.

## Results

### General comments

Among the 63 studies selected for this review, we found that 84% of the articles focused solely on mother-child interactions. This is not surprising given our inclusion criteria. The number of dyads examined in the selected studies varied from 2 [52] to 153 [53] with a mean of approximately 50 dyads (mean (±SD)  = 49.2 (±36.29)). Children in the study had a mean age of approximately 1 year (mean (±SD)  = 15.67 (±18.01) months). Characteristics of the mothers were not always reported even for age or parity, two parameters that have been implicated in quality of parenting [15]. Among studies with exploitable data (n = 27), mean age was nearly 30 years (mean (±SD)  = 29.25 (±3.18) years). Most studies did not distinguish primiparous and multiparous women.

Regarding synchrony, about half of the articles (n = 31) focused primarily on synchrony or a similar concept such as reciprocity. The other half (n = 32) concerned mother-child interactions more globally: 20 of these studies included specific questions about synchrony in their methodology and 12 studies provided data detailing the extent to which child and caregiver were synchronized on specific behaviors in the results section. The characteristics of synchrony have been described in several previous works ([54], [55]; see introduction section); we found several definitions of for synchrony which did not always use the same words and which seemed to vary mainly with respect to the theoretical framework of the authors (e.g., cognitive psychology, developmental psychology, psychoanalysis, interaction and engineering). Our reading of the literature indicated that synchrony includes the following components: (1) *A dyad*: an interactive unit/system; (2) *Mutuality*: the partners are mutually regulated; (3) *Reciprocity*: the partners show reciprocity, adaptation, flexibility, and conformity to each other; (4) *Rhythmicity*: the partners maintain balance in the system; (5) *Harmonious interaction*: mother and infant frequently share or experience similar behavioral states and affect; and (6) *Maintained engagement*: the partners experience prolonged social engagement characterized by mutual attention and turn-taking. Reciprocity, a widely operational construct in clinical field, has a large overlap with the concept of synchrony. The main difference applies to the time scale. Reciprocity implicates by definition a large time scale, whereas synchrony can be applied to both macro and micro time scale (see below).

Synchrony has been studied in two different settings: laboratories and natural settings (2/3 vs. 1/3 of the studies, respectively). In most of the cases, interactions were video-recorded. The mean duration of interactions was 11 (±13.13) minutes. The most common types of interactions were (in decreasing order): free play (n = 36), daily routines (n = 10), structured tasks (e.g., specific order) (n = 9), and experimental settings (e.g., still-face) (n = 7). For the studies that indicated a time-frame (n = 38), two primary time scales were used: 10-seconds (n = 10) and 1-second (n = 10).

To simplify the overall presentation of the literature on synchrony, we first summarize synchrony measurement methods. We distinguished three categories of measurement: (1) global interaction scales; (2) synchrony scales; and (3) micro-coded time-series analyses. In the following two sections, we divided the articles according to the targeted population: 39 articles examined early interactions in typically developing populations, whereas 23 articles examined early interactions in clinical populations.

### Synchrony measurement methods

During the last twenty years, there have been several attempts to measure synchrony during early parent-child interactions using an operational coding system (Table 1). We propose distinguishing between synchrony assessment methods as follows:

**Table 1:** Synchrony measurement methods in early mother-child interaction.

Scale	Principles	Main references
***Globalinteractionscales***		
*Coding Interactive Behavior (CIB)* *Qualitative Ratings for Parent-Child Interaction (QR-PCI)* *Coding System for Mother–child Interactions (CMSCI)*	These 3 tools share similar construction, with subscales dedicated to mother, child and dyad. Each subscale is coded according to a predetermined scale or a rating-point system, based on both quantity and quality of the observed behaviors. Video of the interactions is recorded and coded by trained raters. They include different dyadic subscales. The CIB integrated 5 dyadic subscales (reciprocity, adaptation/regulation, smoothness, restriction, tension); results are presented as an interaction profile with 5 components consisting of parental sensitivity, intrusiveness and limit setting, child involvement, withdrawal and compliance, negative state and dyadic reciprocity. The *CIB* is well-validated and demonstrates good psychometric properties. The QR-PCI is a slight modification of the coding system used in the National Institute of Child Health and Human Development (NICHD) Study of Early Child Care. It only has one dyadic subscale called ‘dyadic mutuality’. The CMSCI has a similar construction with additional dyadic behaviors: affective mutuality/felt security, mutual enjoyment and reciprocal interactions.	Feldman, 1998; Feldman, 2012Owen, 1992; Cox & Cornic, 2003Healey, 2010
*NCAST Feeding and Teaching PCI Scales*	The construct is different from others with child and parent items coded as Yes or No; items are added to provide a score. Half of these items include reciprocal interactions and a separate contingency score is determined for both parent and infant.	Keefe, 1996
*Eight-scale CIB parent-infant synchrony*	Extracted from the CIB scale, this instrument is used to index the central behavioral expression of attuned human caregiving. Codes describe parent's behavioral patterns and the coordination of these behaviors with infant signals (i.e., parents adaptation to infant states, resourcefulness in handling various infant communications, and provision of supportive presence for infant play and exploration).	Abraham, 2014
*Belsky Parent-Child Interaction Coding System*	The instrument contains parent–child scales that are coded minute-by-minute. Scales are grouped into four composite variables and five global scales including the degree of parent–child synchrony. Scores are assigned according to the frequency and intensity of verbal and nonverbal behaviors.	Belsky, 1991; Whipple, 1993
*Infant Caregiver Engagement Phases*	The scale codes mother and infant behaviors separately on a second-by-second basis. Engagement categories varied from most negative to most positive. Ham (2009) collapsed them into the following four categories: negative engagement; withdrawn/avoidant; environment engagement; social engagement. Mother-infant synchrony is based on the first-order correlation between the original mother-infant engagement categories for each second of the interaction.	Cohn & Tronick, 1988; Weinberg & Tronick, 1999
*Rating Scale of Interactional Style (RSIS)*	The scale provides global measures of infant involvement, maternal regulation and adaptation. Coders view the entire session and then rate each item from 1 (low) to 5 (high). Synchrony is examined by means of cross-correlation regressive functions (CCF). The CCF assesses whether a lead-follow relation exists between mother's and infant's time series; if a relation is found, it is determined who the leading or following time series belongs to. Three types of synchrony were identified: mother synchrony with infant (mother's time series synchronized with the infant's), infant synchrony with mother (infant's time series synchronized with the mother's), or mutual synchrony (both series synchronized with one another).	Clark & Seifer, 1983
*Behavior State Coding*	Four mother behavior states are coded including anger/poke, disengage, elicit, and play on a scale of 1 to 4 (negative to positive). Related codes are used to describe the infants' facial and vocal expressions and the direction of gaze during interactions. Cohn et al. (1986) included five behavioral states for the infant (protest, look away, object, attend, and play) whereas Field (1989) proposed four infant joint states shared with mothers: anger-poke/protest, disengage/look away, elicit/attend, and play/play. The mothers' and infants' 3-min free-play segments are coded independently and sequentially second-by-second using software. Spectral and cross-spectral analyses are used to study cyclicity and synchrony of behavior states.	Cohn, 1986
***synchronyscales***		
*Bernieri's scale*	The scale uses a rating form based on core aspects of synchrony: simultaneous movement, tempo similarity, coordination and smoothness. A cover sheet explains what each rating was designed to measure, and judges are told that the rating definitions can be interpreted “loosely and liberally.” Each item is rated on a 9-point Likert scale.	Bernieri, 1988
*Synchrony global Coding System*	This system also uses a 9-point item scale to assign a single code to describe a dyad's synchrony, defined as the dyad's reciprocity, shared affect and mutual focus; it is based on non-verbal communication, child positivity and child negativity.	Skuban, 2006
*Dyadic Mutuality Code (DMC)*	The DMC is composed of six subscales (mutual attention, positive affect, mutual turn- taking, maternal pauses, infant clarity of cues and maternal sensitivity in responsiveness to the infant), which are scored 1 (no-occurrence, negative) or 2 (occurrence, positive). The total possible score ranges from 6 to 12. Scores ranging from 6 to 8 are categorized as low responsivity or low synchrony.	Censullo, 1987; Censullo, 1991; Horowitz et al, 2001
*Taxonomy of interactional synchrony*	This scale follows the DMC construction. However, items are based on objective and measurable observations and include four categories of dyadic measures: physical distance, visual orientation, body orientation and dyadic involvement. A time-sampling procedure is used to code behaviors every 10 sec. Each category is scored from 1 to 4 with lower numbers indicating a more synchronous interaction.	De Mendonça, 2011
*Coding Scheme*	These two scales use a time-sample procedure and code each part of the interaction session. The Coding scheme divides interactions into 30-second segments that are individually rated on a 6-point Likert scale. Low ratings indicate asynchronous interactions and high ratings indicate mutual responsiveness, mutual engagement, shared affect, eye contact, and a balance between the mother and the child in offering and following leads. The rating of each segment is averaged to create a score of Interactional Synchrony (IS).	Mize & Pettit, 1997; Keown & Woodward, 2002
*Rocissano and Yatchmink taxonomy*	The *Rocissano and Yatchmink taxonomy* views synchrony as a measure of a dyad's ability to maintain a shared topic. Videos are segmented into a series of events called “turns”, which are assigned to different categories of mutual focus. Turns are defined in terms of those that do (synchronous) or do not (asynchronous) maintain the partner's previous focus of attention. The number of partner's synchronous and asynchronous turns are then compared.	Rocissano & Yatchmink, 1983; 1984
*Maternal-Infant synchrony scale (MISS)*	This is an observational assessment tool designed to assess synchrony during feeding interactions by investigating a concept similar to synchrony: engagement. Listed behaviors selected from the *Mother Infant Feeding Tool* (MIFT, Brown, 2009) are observed for both mother and infant. Engagement is coded for the dyad. Engagement involves the mother-infant dyad behaving simultaneously on 4 selected behaviors: engaged; infant gaze; infant non-gaze and mother attempt.	Reyna, 2012
***Micro-codedtime-seriesanalyses***		
*Monadic Phase Manual*	This scale quantifies maternal and child behaviors by using first annotations of videos and then assessing a cross-correlation that determines the degree of coherence between the two corresponding time-series.	Feldman, 1997, 2003; Moore, 2004
*Others*	Several other proposals investigating specific items have been proposed and associated with statistical analyses	See text

1. Global interaction scales include 9 instruments that assess infant and mother behaviors during interactions and include dyadic parameters. The following 4 scales integrate dyadic items: the *Coding Interactive Behavior (CIB)* scale ([5] French translated version by [56]); the *Qualitative Ratings for Parent-Child Interaction* scale [57], [58]; the *Coding System for Mother–child Interactions (CMSCI)* [59]; and the *NCAST Feeding and Teaching PCI Scales*. This last instrument was used by Keefe [60] to observe and score maternal-infant synchrony on behaviors associated with feeding. These 4 scales give information about the quality of dyadic interactions, but do not directly refer to “synchrony”. In contrast, the following two scales use the term “synchrony” but vary in their method of assessment: the *eight-scale CIB parent-infant synchrony* [61] and the *Belsky Parent-Child Interaction Coding System* [53] (modified version [62]). Contrary to the quality score given by Abraham's eight-scale CIB, scores on Belsky's scale are assigned based on the frequency and intensity of verbal and nonverbal behaviors. Then, appropriate sequential behaviors between partners are coded as synchronous or asynchronous according to the details of the interaction [63], [64]. The last three scales from the global interaction group assess engagement, involvement, mutual synchrony, and shared states and apply a statistical measure to each partner's results. These three scales include: the *Infant Caregiver Engagement* (ICE) scale [65]; the *Rating Scale of Interactional Style (RSIS)* [66] (modified version [67]); and (3) the *Behavior State Coding* scale [68].

Global interaction scales were used in a variety of observational settings and among children of various ages. Free-play was the most common setting used to observe interactions. The NCAST used a teaching moment procedure while the ICE used a still-face procedure. The CIB and the NCAST can be used to observe feeding, and the Belsky scale can be used in the observation of a daily routine, such as bathing. Global scales included in this review assessed children from birth (CIB) to 48 months (CMSCI) of age.

1. Synchrony scales include various assessment designs. Despite being global scales, they differ from the previous ones by being their core focus on synchrony and the absence of subscores regarding each partner's behaviors. Eight global rating systems assign a global score to the parent-child dyad. *Bernieri's Scale* [46] and the *Synchrony Global Coding System* [69] are based on coders' perceptions and judgments of synchrony and are supplemented by item definitions. These two scales treat synchrony as a global concept. After viewing the entire interaction session, the assessment of synchrony is determined by coders' clinical judgments. Video sessions can also be split into several units, based on listed behaviors or time-sampling. Scores are then compiled or averaged to provide an overall assessment of synchrony. This is the case for the *Dyadic Mutuality Code (DMC)* [70], *The Taxonomy of Interactional Synchrony* [71], the *Coding Scheme* [72] (adapted version [73]), and *The Rocissano and Yatchmink Taxonomy* [74]. The last scale, the *Maternal-Infant Synchrony Scale (MISS)* [75] is an observational assessment tool designed to assess synchrony during parent-child feeding interactions by investigating the similar concept of engagement.
2. Micro-coded time-series analyses are methods based mainly on statistical approaches. The most commonly used method of measuring synchrony involves frequency counts of infant and maternal behaviors. Coders use a list of pre-determined behaviors and divide mother-infant observations into brief units of time for assessment. The *Monadic Phase Manual* proposes quantifying maternal and child behaviors by first annotating the videos and then cross-correlating two corresponding time-series to determine the degree of coherence between the two. Maternal synchrony is indicated by the presence of a positive lead-lag between the mother's and infant's time-series [76], [77], or by a correlation between the second-by-second affective state codes given to infants and mothers [78]. Coding is generally assisted by a software system such as The Observer or Elan. Mother-infant synchrony is quantified by the first-order correlation between mother-infant targeted behaviors for every few seconds of interaction [79]. Synchrony may also be assessed by calculating proportions, frequencies, mean durations and latencies of specific relational behaviors [45], [80] or by measuring the correlation between partners' behaviors [81]. Another proposal was to measure synchrony as the percentage of time during which the mother and infant looked at each other's head simultaneously [82]. An example of a frequency score is summing the number of attempts a mother and child make to engage to each other. Similarly, Isabella [83] gathered information on the frequency of a more limited set of maternal, infant and dyadic behaviors by observing mother-infant interactions over successive 15-second intervals. To assess the mean duration of synchrony, Gratier [84] analyzed acoustic segments of interaction. A spectrogram – a visual representation of an acoustic signal – and pitch plot provided information regarding onset, end time, and duration of vocalizations and periods of silence between them; energy; loudness; and the pitch of acoustic signals. Groups of vocalizations bounded by pauses lasting more than 500 milliseconds were called “phrase units”. Expressive timing refers to the degree of variation from a strictly regular “pulse” or “beat”. Interactions were coded as “interactional synchrony” when vocalizations occurred simultaneously (overlap), were successive (turn-taking) or were imitative, with respect to matching pitch and rhythm.

### Studies investigating synchrony in normal populations

Table 2 summarizes the main characteristics and findings for the studies investigating synchrony during early parent-child interactions in normal populations. Articles were sorted into 6 categories: validation of synchrony assessment tools (n = 6); variation in age and gender (n = 7); variation between parents (n = 6); micro-interest (synchrony is observed for a specific behavioral modality; n = 12); associations with physiological data (n = 5); and other (n = 3).

**Table 2:** Main characteristics and findings of the studies investigating synchrony in normal populations.

Author (year)	Study design	N	Mother age (years)	Mother SES	Mother ethn^icity^	Primi/multi^para^	Infant age (months)	Assess^ment^ tools	Main findings
**Validation of synchrony assessment tools**									
**Bernieri (1988)**	Paired comparison: Genuine synchrony *vs*. pseudo-synchrony	8	dnf	dnf	dnf	Both	14–18	Movements	Genuine synchrony was higher than pseudo-synchrony; mothers with unfamiliar children demonstrated a state of dissynchrony
**Booth (1984)**	Longitudinal case-series	56	dnf	lower-middle class	dnf	Dnf	10–12	Behaviors and regular activities	The frequency-based synchrony measure developed in this study is stable across the 2 observations. It better reflected the dyad as a system and demonstrated more stability than the duration measure. Relationships were found between the synchrony score and scores on the HOME Observation for Measurement of the Environment.
**Censullo (1987)**	Validity study. Paired comparison: preterm *vs*. full term	38	18–35	dnf	White	Dnf	dnf	Dyadic Mini Code (DMC)	Construct validity was demonstrated through a report of significant differences between pre-term and full-term infants on the synchronous subscale
**Reyna (2012)**	Descriptive longitudinal study	10	20–42	High-school & college	Cauc^asian^ (80%)	Dnf	Birth, 1 then 4	Maternal Infant Synchrony Scale (MISS)	The synchrony tool developed in this study demonstrates that changes occur in mother and infant behaviors over time. Infants' attempts at interaction were greater than mothers' attempts to engage with their infants
**Viaux (2014)**	Translation validation of an assessment scale	37	32	Bach^elor^ degree (72%)	dnf	Both	1	Coding Interactive Behavior (CIB)	Inter-rater reliability of the training was good with a median ICC equal to 0.88. Confirmatory factor analyses supported a comparable latent factor structure as reported in the original CIB with α ranging from 0.67 to 0.96 at birth and 0.63 to 0.95 at 2 months. Inter-rater reliabilities at birth and 2 months were good, with ICCs ranging from 0.85 to 1 for each item
**Tronick (1977)**	Descriptive study	3	Dnf	Dnf	Cauc^asian^	Both	2	Vocalization, facial expression, touch, body/head position	Results show the occurrence of long periods of interaction during which the quality of the infants' displays approached an almost perfect synchrony with that of the mother. The smooth synchronous flow of the mother–infant interactions indicated that the infants are able to communicate intents and to respond to the intent expressed by their mothers
**Variations in children age**									
**Feldman (1996)**	Longitudinal case-series study	36	28.7±1.5	Middle-class	Israely	Both	3 then 9	Attentive-affective states (from positive to negative engage^ment^)	Synchrony was higher at 9 months than 3 months. At 3 months, mother synchrony with the infant predicted visual IQ at 2 yrs.
**Feldman (1997)**	Longitudinal prospective case-series study	36	28.7±2.5	Middle-class	Israely	Both	3 then 9	Affective states	Maternal synchrony at 3 and 9 months had an independent contribution to the prediction of symbolic play and internal state talk at 2 yrs.
**Feldman (2003)**	Correlational study	100	27.7±3.93	Middle-class	Israely	Primi^para^	5	Facial expression, vocalization, gaze, body orientation, level of arousal	Synchrony between same-gender parent–infant dyads exhibited more frequent mutual synchrony. Mother-infant synchrony was linked to the partners' social orientation and was inversely related to maternal depression and infant negative emotionality. Father-infant synchrony was related to the intensity of positive arousal and to father attachment security.
**Feldman (2004)**	3 paired comparisons: singleton *vs*. twins *vs*. triplets	138	28.5	Middle-class	Dnf	Multi^para^	3 and 12	Gaze, vocalization, touch	Lower parent-infant synchrony was observed for triplets. Dyads showed reduced capacities to coordinate social behaviors in the different modalities into a well-matched synchronous dialogue. Behavior problems were predicted by parent infant synchrony
**Feldman (2007)**	Longitudinal case-series study	31	28.7±2.5	Middle-class	Israely	Both	3 then 9	Affective involvement	Mother-infant synchrony predicted verbal IQ and behavioral adaptation. A significant association was found between synchrony and the capacity for empathy in adolescence.
**Howe (2001)**	Longitudinal, prospective study	32	Dnf	Middle-class	Cauc^asian^	Multi^para^	14 then 85	List of behaviors (child engage^ment^, hostility, parental manage^ment^)	Poor synchrony between maternal directive management and sibling hostility was associated with less sibling cooperation over time.
**Tronick (1989)**	Paired comparison according to age	54	Dnf	Dnf	Cauc^asian^	Both	3, 6 then 9	Body/head position/movement, facial expression, gaze, vocalization	Mother-infant pairs increased their degree of coordination with infant age, but the proportion of time for which they were coordinated was small. Mother-son pairs spent more time in coordinated states than mother-daughter pairs. The results suggest that interactions may be characterized in terms of their movement from coordinated to uncoordinated states rather than only in terms of their degree of coordination.
**Variation between Parent**									
**Abraham (2014)**	Paired comparison: Primary-Caregiving (PC) mothers *vs*. Secondary caregiving fathers *vs*. PC homosexual fathers	89	36.1±4.54	Dnf	dnf	Primi^para^	11	8-scales CIB parent-infant synchrony: Affect, gaze, touch, vocalization, parent's adaptation	No inherent differences in the parental caregiving context as a function of the parent's sexual orientation.
**De Mendonca (2011)**	Paired comparison: Father *vs*. mother	42	dnf	Middle-class	French Cana^dian^	Dnf	32	Physical proximity; body/visual orientation; dyadic involvement	Similar patterns of interactional synchrony (IS) in mother-child and father-child dyads in dyadic context. Father-child dyads presented less IS than mother-child dyads when they interacted in triads.
**Dubrow (1999)**	Paired comparison: Mother *vs*. father; Younger *vs*. older siblings	30	dnf	Middle-class	Cauc^asian^	Multi^para^	24 to 108	Physical and verbal behaviors.	There is a greater degree of synchrony or reciprocity in the dyadic relationship between mothers and siblings than between fathers and siblings. Specifically, siblings' behavior directed toward the mother correlated significantly with maternal behavior toward the children.
**Garcia-Sellers (2000)**	Paired comparison: mother *vs*. father	26	dnf	Dnf	Dnf	Both	21	Dnf	For mothers, synchrony in interactions most strongly predicted internalizing behavior, whereas for fathers, focus on control and direction style predominated.
**Gordon (2008)**	Paired comparison: (co-parenting, Father/mother)	94	27.7±3.93	Middle-class	Israely	Primi^para^	5	Gaze, affect, object manipula^tion^, vocalization, touch	Mothers' and fathers' relational behavior were generally comparable, yet mothers vocalized more and the latency to father's displaying positive affect was longer. Under conditions of coparental mutuality, fathers showed more positive behaviors than mothers. Fathers' coparental mutuality was independently predicted by maternal behavior during mother-child episodes, father marital satisfaction, and infants' difficult temperament, whereas mothers' coparental mutuality was only associated with fathers' relational behavior.
**Lundy (2003)**	Paired comparison: Mother *vs.* father	48	26.79±4.63	Lower-middle class	Cauc^asian^ (96%)	Both	6	Belsky Parent Child Interaction Coding System	For both mother and father, frequency of interactional synchrony was found to mediate the relation between mind-related comments and attachment security
**Micro-interest (gaze, smile…)**									
**De Graad (2012)**	Longitudinal descriptive study	84	32.5±3.9	Middle/upper-class	Dnf	Primi^para^	5	Gaze	Infant sleep could predict the temporal dynamics of the mother-infant interaction (flexibility of gaze), but did not predict the percentage of synchrony.
**Dowd (1986)**	Descriptive study	6	dnf	Dnf	Cauc^asian^	Dnf	0.5–2.5	Arm movements	No evidence was found for interdependence between right and left arm movements, or between speech events and arm movements
**Farran (1990)**	Controlled trial	42	dnf	10th grade	Black	Multi^para^	6, 20 then 36	Gaze	Duration and frequency of mutual gaze increased over months. Mothers initiated the majority of mutual looks but mothers' initiation decreased over time, while children initiated more mutual gaze. Mothers who had been assigned to a day-care program had a higher proportion of mutual regards than the control group.
**Gogate (2013)**	Correlational study in a cross-over design	42	dnf	Middle-class	Hisp^anic^ & cauc^asian^	Dnf	6–8	Object naming object motion	To further highlight object wholes during naming, mothers predominantly shook or loomed object wholes in synchrony with their naming more often than in synchrony with object parts.
**Gratier (2003)**	Paired comparison: India *vs.* US *vs.* France *vs.* immigrant	60	dnf	Dnf Paired SES	Mixed	Primi^para^	2–5	Vocal interactions	The 3 cultural contexts manifested the same interactional synchrony in vocal interactions. The immigrant dyads showed lower levels of interactional synchrony than the non-immigrant dyads.
**Longhi (2009)**	Descriptive study	4	dnf	Dnf	Dnf	Both	3–4 and 7–8	Non-verbal behaviors	Mothers' singing and synchronous behaviors with the beat revealed that mothers emphasized the hierarchical structure of the song and provided a segmentation of the temporal structure of the interaction. Infants indicated sensitivity to their mothers' emphasis by producing significantly more synchronous behaviors on some beats than on others.
**Matatyaho (2008)**	Correlational study	24	dnf	Dnf	Mixed	Dnf	7	Object motion and naming	Maternal use of shaking motions in synchrony with spoken words and infants' ability to switch gaze from mother to object contributed to infants' learning of the word–object relations.
**Messinger (2009)**	Descriptive study	2	dnf	4 years of college	White	Dnf	6	Smiling	For both mothers and infants, smile strength and eye constriction (the Duchenne marker) were correlated over time. Infant and mother smile activity exhibited changing (non-stationary) local patterns of association, suggesting the dyadic repair and dissolution of states of affective synchrony.
**Rocissano (1987)**	Descriptive study	10	dnf	middle-income	Dnf	Primi^para^	16–20	Rocissano and Yatchmink taxonomy	Children were most likely to take synchronous turns directly following maternal synchronous turns. Synchrony was positively correlated with child compliance. Children were more likely to comply with synchronous caregiver instructions than with asynchronous instructions.
**Roe (1997)**	Descriptive study	23	dnf	All ranges	Amer^ican^ & Greek	Primi^para^	3	Frequency of vocalizations	Mothers in the mid-level talking range demonstrated the greatest reciprocity, allowing their infants to initiate more conversations. The most talkative mothers did not allow their infants to initiate many conversations. The least talkative mothers ignored many of their infants' vocalizations.
**Van Puyvelde (2010)**	Descriptive study	15	28±4.5	Middle-class	Dutch	Primi^para^	3	Vocal exchanges	The total duration of dyads being in tonal synchrony was normally distributed. Tonal synchrony and its characteristics are discussed in relation to infant-directed speech, communicative musicality, pre-reflective communication and its impact on the quality of early mother–infant interactions and child's development.
**Physiological markers**									
**Ham (2009)**	Correlational study. Still-face paradigm. Vagal tone	18	33±5	dnf	Mixed	Dnf	5	Gaze; Vocalization; Facial expressions	During reunion episodes, when mothers soothe their infants, skin conductance is correlated with behavioral synchrony. Behavioral synchrony is strongly related to infant respiratory sinus arrhythmia (RSA).
**Gordon (2010)**	Prospective study. Hormonal correlates	37	26.26±3.94	Middle-class	Dnf	Primi^para^	6	Proximity, touch, gaze	Triadic synchrony was predicted by both maternal and paternal oxytocin. Triadic synchrony was independently related to lower levels of maternal cortisol.
**Moore (2004)**	Descriptive study. Still-face paradigm. Vagal tone	73	29.1±5.44	Middle/upper-class	Cauc^asian^ (81%)	Dnf	3	Facial affect, direction of gaze	Infants who did not suppress vagal tone during still-face (non-suppressors) showed less positive affect and lower synchrony in normal play with mothers. The results indicate that infant's physiological regulation in social interactions differs in relation to dyadic coordination of affective behaviors.
**Penman (1983)**	Correlational study. Infant neurophysiological capacities and interaction	10	17–30	dnf	Dnf	Both	3	Engagement – disenga^gement^	Significant correlations were found between neonatal responsivity and motor maturity and the nature of mother-infant interactions at 3 mo. Infants who were more socially responsive and attentive to stimuli had mothers with a greater capacity for screening-out redundant stimuli. These dyads spent more time in social engagement and had fewer cycles of disengagement.
**Thompson (2009)**	Correlational study Infant cognition	94	24.8±6.5	dnf	Cauc^asian^/Hisp^anic^	Primi^para^	6	Belsky's listed behaviors (including gaze, vocalization)	Maternal sensitivity, as reflected in higher mother–infant behavioral synchrony scores impacts infants' cognitive processing of mother-specific face patterns.
**Other**									
**Harrist (1994)**	Prospective study	dnf	dnf	dnf	Dnf	Dnf	60	Dyadic synchrony	Positive school adjustment was predicted by high levels of positive synchrony, low levels of non-synchrony, and low levels of negative synchrony in mother-child interactions.
**Lindsey (1997)**	Correlational study	35	dnf	dnf	Dnf	Both	45–76	Attention, social competence	More synchronous mother–child and father–child dyads had higher mutual initiation and mutual compliance scores
**Tronick (1986)**	Correlational study	18	dnf	dnf	Cauc^asian^	Dnf	3, 6 and 9	Coping behaviors for repairing interactive mismatches	For about 70% of the time, mothers and infants were not in matching stage or in synchrony. Normal infants experience a large number of mismatches in normal interactions with adults. Infants have coping behaviors for repairing the interactive mismatches and maintaining self-regulation that are largely successful and that begin to stabilize at 6 months of age

dnf: Data not found. SES: Socio-economic status.

The main results can be summarized as follows: (1) Synchrony during early mother-child interactions has neurophysiological correlates [85] as evidenced though the study of vagal tone [78], cortisol levels [80], and skin conductance [79]; (2) Synchrony impacts infant's cognitive processing [64], school adjustment [86], learning of word-object relations [87], naming of object wholes more than object parts [88]; and IQ [67], [89]; (3) Synchrony is correlated with and/or predicts better adaptation overall (e.g., the capacity for empathy in adolescence [89]; symbolic play and internal state speech [77]; the relation between mind-related comments and attachment security [90], [91]; and mutual initiation and mutual compliance [74], [92]); (3) Lack of synchrony is related to at risk individuals and/or temperamental difficulties such as home observation in identifying problem dyads [93], as well as mother-reported internalizing behaviors [94]; (4) Synchrony has been observable within several behavioral or sensorial modalities: smile strength and eye constriction [52]; tonal and temporal analysis of vocal interactions [95] (although, the association between vocal interactions and synchrony differs between immigrant (lower synchrony) and non-immigrant groups [84]); mutual gaze [96]; and coordinated movements [37]; (5) Each partner (including the infant) appears to play a role in restoring synchrony during interactions: children have coping behaviors for repairing interactive mismatches [97]; and infants are able to communicate intent and to respond to the intent expressed by the mother at the age of 2 months [98]. Additionally, children are sensitive to synchronous parental behaviors such as maternal synchronous turn-taking and giving of instructions [74]; and (6) Synchrony also depends on parental characteristics and/or skills such as maternal sensitivity [64], [99]. Although synchrony shows similar patterns within the mother and father dyadic contexts, father-child interactions (compared to mother-child interactions) exhibit less synchrony in the triadic context [71]. Moreover, no inherent differences are found in the parental caregiving context as a function of the parent's sexual orientation [61]. In general, same-gender parent-infant dyads seem to experience more synchrony [76], however one paper found that mother-daughter dyads spend less time in coordinated states compared to mother-son dyads [37]. Mothers interacting with an unfamiliar child show less synchrony [46]. Lower parent-infant synchrony was observed among triplets compared to twins or singletons [100]. Finally, it appears that synchrony is not an uninterrupted process, as changes in synchrony occur over time [51]. Yet, even if dyads increase in their degree of coordination over time, the proportion of time they are synchronous remains small. These results suggest that interactions may be characterized both in terms of movement from coordinated to uncoordinated states as well as the degree of coordination during interactions [37].

### Studies investigating synchrony in clinical populations

Table 3 summarizes the main characteristics and findings of the studies investigating synchrony during early parent-child interactions in clinical populations (n = 33). We found 12 studies investigating infant psychopathology or developmental impairments, 6 studies comparing mother-child interactions among normal control mothers compared to mothers presenting with a mental or medical condition, and 5 studies investigating the subtypes of child attachment styles.

**Table 3:** Main characteristics and findings of the studies investigating synchrony in clinical population.

Author (year)	Study design	N	Moth^er^ age (years)	Mother SES	Mother ethn^icity^	Primi/multi^para^	Infant age (months)	Assessment modalities	Main findings
**Infant psychopathology**									
**Ambrose (2013)**	Correlation study. Physical and relational aggression	73	Dnf	Middle-class (58%)	Cauc^asian^(76%)	Dnf	36–72	Coding scheme	Dyadic synchrony between mother and child is related to the level of child functioning, above and beyond the influence of symptom severity. High dyadic synchrony plays a protective role and leads to better functioning in pre-schoolers displaying elevated levels of hyperactivity/inattention.
**Bonaminio (1983)**	Paired comparison. Autism *vs.* typical	dnf	Dnf	Dnf	Dnf	Both	12	Dnf	An integrated model of normal and autistic mother–infant relationships is suggested in which the autistic disturbance is viewed as a deep alteration of rhythms, synchrony, and reciprocity between mother and infant.
**Harel (2011)**	Paired comparison Pre-term *vs.* full term	60	31	Middle-class	Dnf	Both	3	Gaze	Preterm infants and their mothers displayed short and frequent episodes of gaze synchrony. Both mothers and infants broke moments of mutual gaze within 2 sec of its initiation. Proportion of look away during behavior response paradigm was related to lower gaze synchrony
**Healey et al (2010)**	Correlation study Hyperactive-Inattentive scores	126	Dnf	Middle-class	White non-hispanic (40%)	Dnf	36–48	Coding System for Mother-Child Interactions (CSMCI)	Dyadic synchrony between mother and child is related to the level of child functioning, above and beyond the influence of symptom severity. High dyadic synchrony plays a protective role and leads to better functioning in pre-schoolers displaying elevated levels of hyperactivity/inattention.
**Karger (1979)**	Paired comparison Pre-term *vs.* full term	49	23±7	Lower-class	Black	Multi^para^	3 and 9	Communicative behaviors	At 3 months, measure of synchrony differ between Full Term (FT) and Pre Term (PT). PT: less mother and infant responsivity. NSP: maternal tendency to persistently respond in the absence of infant responding. PSP: mothers tend to refrain from responding when their infants become non attentive. No correlation with HOME scores.
**Keefe (1996)**	Paired comparison Irritable *vs.* non irritable	40	Dnf	Dnf	Dnf	Both	2–4	Nursing Child Assessment Satellite Training (NCAST)	Irritable children showed less synchrony in mother-infant interaction
**Kirsh (1995)**	Longitudinal paired comparison Preterm *vs.* full term	69	Dnf	Dnf	Dnf	Both	60	Dnf	Synchrony and affect predicted lower cognitive abilities
**Lester (1985)**	Paired comparison Preterm *vs.* full term	40	Dnf	Matched for SES	Cauc^asian^	Dnf	3 and 5	The Monadic Scale	Term dyads showed higher coherence than preterm dyads at both 3 and 5 months. Term infants more often led the interaction at both ages.
**Ravn (2011)**	controlled trial moderately and late preterm	93	Dnf	Dnf	Norwegian	Dnf	12	Qualitative Ratings for Parent-Child interaction	Being a first-time mother was a moderator that enhanced the effects of the intervention. First-time mothers were more sensitive/responsive to their infant's cues (p = .01), and the dyads evinced higher level of synchrony (p = .02) as compared with experienced mothers.
**Shapiro (1987)**	Exploratory study Autism *vs.* dysphasia	6	Dnf	Dnf	Dnf	Both	32–43	Communicative interaction	Results show that mothers of these children were less able to set up successful dialogues because they frequently redirected them. After a nursery program of 5 to 8 months, mothers became less asynchronous.
**Skuban (2006)**	High-risk, low income toddler boys	120	27.2±6.1	Lower- middle-class	Black, Cauc^asian^, Biracial	Both	24	Synchrony Global coding System	Synchrony was associated with aspects of parenting and child attributes, including maternal nurturance, and child emotional negativity and language skills. The findings are discussed in terms of parent and child contributions to the development of synchrony.
**Venuti (2009)**	Paired comparison Down Syndrome *vs.* typical	54	36±5.79	Lower- middle-class	dnf	Both	dnf	Solitary and collaborative mother-child play	Both groups showed similar attunement and synchrony. *mothers contribute to the play development of children with Down Syndrome through their own adaptation to their children's limitations and potentialities.
**Parent**									
**Field (1989)**	Paired comparison: Depressed *vs.* non-depressed	16	26	Lower-class	Black	Primi^para^	3	Behavior State Coding	The depressed mothers and their infants shared negative affective behavior states more often and positive behavior states less often than the non-depressed dyads.
**Field (1990)**	Paired comparison: Depressed *vs.* non-depressed	48	27	Lower-class	Black	Both	3	Behavior State Coding	Cross-spectral analyses of the mothers' and the infants' behaviour-state time series suggested only a trend for greater coherence or synchrony in the interactions of the non-depressed dyads
**Gandillot (2012)**	Paired comparison: Depressed *vs.* psychotic	18	32.7±6.52	Dnf	French Cauc^asian^	Dnf	2,5	Belsky Parent Child Interaction Coding System	Maternal psychopathology, either depressive or psychotic, is associated with dysharmonious interactive styles. The main common characteristics are lack of attunement and scarcity of interactive exchanges. Maternal depression seems to have more influence on interaction synchrony and on both partners' withdrawal.
**Rosenblum (1997)**	Paired comparison: Depressed *vs.* non-depressed	54	dnf	Dnf	French Cauc^asian^ (52%)	Both	12, 18, 24 and 36	Affective Involvement State Scale (AIS)	Anxious and stressed depressed mothers maintained a low level, but synchronic, affective involvement with their one-year-old infants who characteristically showed an insecure-ambivalent attachment to their mother.
**Weinberg (2006)**	Paired comparison: High symptom *vs.* low symptom *vs.* control group. Still face paradigm	133	24–40	Middle-class	dnf	Both	3	Facial expression	Male as compared to female infants were more vulnerable to high levels of maternal depressive symptoms. High symptom mothers and their sons had more difficult interactions in the challenging reunion episode.
**Zlochower (1996)**	Paired comparison:Depressed *vs.* non-depressed	35	dnf	Middle-class	Cauc^asian^	Dnf	4	Speech signal	Switching pauses of depressed mothers were longer, more variable, and less consistent with scalar timing. Depressed mothers in a low-risk population were less responsive to their 4-month-old infants and used a timing mechanism that was less predictable.
**Dyad attachment**									
**Crandell (1997)**	Paired comparison: Insecure vs. secure	36	33±4	Middle-class (72%)	Cauc^asian^	Dnf	35–46	Belsky Parent Child Interaction Coding System modified version	Degree of dyadic synchrony was related to the quality of maternal representations of attachment relationships (the most striking differences between the 2 groups) = SECURE: more responsive to each other's cues and engaged in a more fluid process of give and take
**Isabella (1989)**	Longitudinal paired comparison: Secure *vs.* avoidant *vs.* resistant	30	dnf	Middle-class	Cauc^asian^	Primi^para^	1, 3 and 9	Belsky Parent Child Interaction Coding System	At 1 and 3 months, development of secure attachment was predictable from synchronous interactions. In contrast, insecure attachment was predictable from asynchronous
**Isabella (1991)**	Paired comparison: Insecure *vs.* secure	153	26.8	Middle- class	Cauc^asian^	Primi^para^	3 and 9	Belsky Parent Child Interaction Coding System	At 3 and 9 months, dyads developing secure attachment showed well-timed, reciprocal and mutually rewarding interaction; Resistant attachment style dyads showed poorly coordinated interaction in which mothers were under involved and inconsistent.
**Lundy (2002)**	Multidimensional comparison: (i) Father *vs.* Mother (ii) Attachment style (iii) Depression *vs.* no depression	30	27.87±4.27	Lower-middle class	Cauc^asian^(96%)	Both	6.3	Belsky Parent Child Interaction Coding System	Synchrony accounts for the relation between marital quality and infant attachment to fathers, and between depressive symptoms and level of attachment to mothers.
**Moss (1997)**	Paired comparison:(i) Insecure *vs.* secure (ii) Stranger *vs.*mother	27	30	Middle-class	French Cana^dian^	Both Primi^para^ (70%)	43	dnf	The collaborative style of mothers of secure children showed more synchrony with secure children's level of participation in the task than with that of insecure children who were less focused on goal-directed task activities

dnf: Data not found. SES: Socio-economic status.

The main study objectives were to describe and/or evaluate parent-child interactions through micro-interest or validation of synchrony assessment tools; to compare the quality of interactions according to infants' characteristics: term *vs.* pre-term or typical development *vs.* pathology (aggressive behavior; ADHD; Down syndrome; autism); and to compare the quality of interactions among parents experiencing pathology (depression; psychosis) *vs.* healthy controls.

The main results can be summarized as follows: (1) Among children with externalizing behaviors, synchrony is associated with the level of child functioning and plays a protective role in the development of ADHD [59]; the association between externalizing behaviors and synchrony is not gender dependent [59]. Lower levels of synchrony were found during early interactions among parent-child dyads with children who had higher levels of parent-rated physical aggression [101] and infant irritability [60]; (2) Among pre-term infants, authors found lower coherence during interactions led by the infants [102], less mother and infant responsivity [81] and shorter episodes of gaze synchrony [103]. Additionally, lower cognitive abilities were correlated with lower levels of synchrony [104]; (3) Whereas no differences in synchrony were found during early parent-child interactions among children with Down syndrome compared to typically developing children [105], synchrony was lower among children with autism [106]. Participation in a nursery program was shown to improve synchrony among parent-child dyads where the child had autism [107]; (4) Among high-risk, low-income, toddler boys, synchrony was positively associated with maternal nurturance and language skills, and negatively associated with child emotional negativity [69]. First-time mothers who engaged in a therapeutic program evinced higher levels of synchrony [108]; (5) Studies with depressed mothers found that more negative and less positive affective behaviors were shared during mother-infant interactions [68], a trend which corresponded with less synchrony/coherence [109]. Males with depressed mothers appeared to be more vulnerable than females with depressed mothers [110]. Additionally, with respect to vocalizations, depressed mothers were less responsive and predictable [111]. As with depressed mothers, authors found less synchronous parent-child interactions among psychotic mothers [63]; and (6) In terms of attachment styles, synchrony during interactions (high *vs.* low) predicted children's profiles (secure *vs.* insecure) [53], [83]. Synchrony was also related to the quality of maternal representations of attachment relationships [109].

## Discussion

### Summarizing the results

Whatever the assessment method, it appears that synchrony should be regarded as a social signal *per se* as it has been shown to be a valid concept in both normal and pathological populations. Better mother-child synchrony is associated with familiarity (vs. unknown partner), a healthy mother (vs. pathological mother), typical development (vs. psychopathological development), and more positive child cognitive and behavioral outcomes. Within normal populations, studies have shown that synchrony varies developmentally, mirroring children's communicative abilities that allow them to be increasingly interactive with their first caregivers and others [67], [88], [97], [112], [113]. During the first year of life, synchrony is often intermodal, characterized by a mother's voice and a child's movements, for example. As children get older, synchrony may be characterized by more symmetric modalities, increased child initiation and turn-taking. A synchronous interaction may not be interpreted as a perfect symmetric timing exchange. Breaks and variations are important to improving adaptation, creativity and stimulation.

Synchrony is also a criterion for distinguishing between normal and pathologic interactions. In the case of maternal pathologies that have been extensively investigated (e.g., depression), mother-child interactions demonstrate lower levels of synchrony [110], [114]. More generally, interactions are poorer each time one of the partners is impeded by internal (pathological) or environmental distractor. As such, parent-child interaction may be poorer when maternal sensitivity and empathy is impeded (i.e., during maternal depression) or when child pathology does not permit a child to answer his mother. From the view of studying synchrony, it appears that there is an interest in studying clinical populations, not with respect to specific symptomatology, but rather more broadly as an indicator of a maternal or child trait that may signal a reduced capacity to be interactive. Synchrony is not an all or nothing concept; rather, it may be more valid to think about dyadic interactions as approaching or moving away from synchrony [3]. Additionally, all studies that focus on child development demonstrate a link between synchrony and attachment, on one hand [83], and child cognitive and behavioral development, on the other [26], [89].

### Limitations

Given our study design focusing on synchrony in the context of early interaction, the current review cannot be considered as an exhaustive review. We decided to focus on mother-child because this dyad has been the most closely examined for synchrony. Interacting studies relative to synchrony may not be reviewed by our methodology. However, because of our inclusion criteria some interesting studies about specific dyad [115]–[117] were not included. Additionally, given the number of concepts close to synchrony, we had to limit them to keep focusing on synchrony only. In consequence, the Care-index [118] was not included in our review, even if it proved its validation. The Care-index is a qualitative scale that has been used widely. However, it does not allow synchrony assessment and rather gives affective tone of the interaction, interactive style and strategies of each partner. It is especially based on turn-taking.

Also, interpretation of the studies may be related to inerrant limitations of the different methods that were used. First, as exposed in the synchrony measurement section and discussed in section 4.3, many scales were not properly validated. Second, one major difficulty in measuring synchrony is identifying and defining critical behaviors that are specific enough to capture the variations in individual behavior yet broad enough to account for maturational changes. Some behavior can be considered positive or negative depending on the frequency of occurrence [3]. Third, another difficulty is to determine interaction rhythm and to order initiations and break out. The video recording of mother-infant interaction poses certain technical challenges. The angle of the camera has to be set so that the mother and infant could be seen clearly. Behaviors may have changed due to the influence by the observation camera and the chosen setting [51]. Fourth, besides definition of synchrony construct and quality of clinical tools and recording, a striking observation is that many studies neglected to properly describe and take into account mother characteristics such as age, parity, socio-economic status or ethnicity (see table 2 and 3). Future studies should improve mother characteristics reporting.

Finally, given the heterogeneity of the studies' definitions and methods, we cannot exclude that our review methods based on consensus may introduced biases in the result synthesis. We consider this work as a starting point for fruitful discussion among the field and across disciplines.

### Comments on definition and assessment methods

The main objectives of the present review were to understand how synchrony in early mother-child interactions has been defined and measured in the literature and to determine which contributions the concept of synchrony increase our understanding of the nature of mother-child interactions. The results revealed differences in the theoretical backgrounds and the methodological assessments used to study synchrony. We distinguished three different types of assessment tools. The first type are global interaction scales which include a dyadic assessment (sometimes clearly called synchrony) and provide a global and qualitative description of interaction. The second type are specific synchrony scales. Some synchrony scales are specifically constructed for one study and therefore were not externally validated (e.g. [69]). Some synchrony scales are global and qualitative while others are constructed with listed behaviors which allow for the assessment of synchrony using statistical measurements (e.g., frequency, duration, co-occurrence). Similar to the global scales, the specific scales do not always use the term synchrony in the assessment tool. The third type of assessment tool includes evaluation and statistical tools which describe synchrony on specific behaviors and focus on the temporal component of parent-child interactions. In sum, it appears that the lack of common evaluation methods in the study of synchrony may introduce bias in the interpretation and comparison of study results between studies.

Studies investigating synchrony in early interactions include closed concepts such as reciprocity [23], [53], [101], [119], [120], shared affect [23], [121], attunement or mutuality [70], [79], [98], rhythmicity [83], [84], [86], [102], [122], harmonious interaction [121], [123], [124] and maintained engagement [82], [97]. These components appear to be relevant to the assessment of synchrony. However, examination of the dyad system and its associated interactional behaviors along with temporal patterns may be the most relevant way to measure synchrony. Yet, the challenge of finding an objective and shared method of measuring synchrony remains. The measurement of synchrony should not be dependent on the number of parent-child interactions but rather on the number of interaction attempts compared to successes. Moreover, an effective measure of synchrony should differentiate between initiated behavior and response behavior [11]. Even if the method is grounded by specific behaviors, what remains important is the sequence or pattern of these behaviors and their relationship to each other within the dyadic exchange [51]. A useful measure of synchrony should also control for any bias on the part of the observer. Moreover, it should allow for individual differences. It is important to refer to a synchronous *interaction* rather than a synchronous *relationship*, because the observable patterns of dyadic interactions provide a “window” to the social relationship of the interacting partners [72], [125]–[127]. The number of concepts related to synchrony further suggests that interactions may be best characterized in terms of their movement from coordinated to uncoordinated states rather than solely in terms of their degree of coordination during interactions [37]. We can therefore define synchrony, inspired by Delaherche [34], as the dynamic and reciprocal adaptation of the temporal structure of behaviors and the sharing of affect between interactive partners.

### Proposals to improve assessment methods

Synchrony is a complex phenomenon requiring the perception and integration of multimodal communicative signals. It has not only been investigated in the field of early parent-child interactions and developmental psychology but also in the fields of social psychology, neuroscience [27], engineering, and robotics [34], [47]. We propose that combining several approaches within a multidisciplinary perspective at the intersection of social signal processing, computational neuroscience, developmental psychology and child psychiatry may efficiently address some of the challenges faced in better understanding synchrony in terms of its neurophysiological and psychological correlates [48]. By providing automatic, detailed and objective measures of multimodal socio-emotional behaviors, we believe that our proposal will become a valuable tool for examining early language, emotional and social interactions in both normal and clinical populations.

In the field of autism spectrum disorders (ASDs), we already applied this approach with some novel findings. When studying home movies with computational methods combined with behavioral annotation and temporal synchrony, we demonstrated the following: (1) infants who will develop autism could be differentiated from both infants with intellectual disabilities and typically developing (TD) children as early as the first year of life [11]; (2) parents adapted the way they interacted with infants who will develop autism by being more supportive and by using more parentese [13], [128]; (3) fathers of infants developing autism spoke to their infants more than fathers of TD infants during the 12–18 months period [13]. To conduct these studies, we first developed an automatic computerized tool to differentiate parentese vs. normal speech [129]. Other studies using a prospective approach in at risk samples also found results highlighting the importance of emotional synchrony and caregiver adaptation to infant lack of inter subjective behaviors when developing autism. Recently, the British Autism Study of Infants' Siblings reported that early dyadic interaction between at-risk infants and their parents was associated with later diagnosis of autism [14]. Also, in infants with West syndrome (an early onset epileptic encephalopathy with high risk of autism during outcome), the lack of synchronic interaction during the first year of life predicted those who will develop autism and intellectual disability [130]. Together, with the studies summarized above [106], [107], all these evidences suggest that impaired parent-infant interaction during the first year of life may be an early marker of autism.

Aside from natural settings (e.g., home movies), this approach can also be applied in the laboratory as well as more experimental settings. By studying motion with computational methods, Weisman et al. [131] showed that oxytocin shaped parental motion during early infant-parent interactions. Similarly, using a specific algorithm for speech turn tracking (STT) during a still-face experiment with typically developing infants, Weisman et al. [132] found the following: (i) infant vocalization and STT were key social cues used to regulate interactions during still-face and during reunions after still-face, with infant vocalizations leading interaction dynamics; (ii) father pause (more so than father vocalization or fatherese) was the main adaptive behavior for fathers after still-face; (iii) oxytocin did not modulate infant STT or father STT/fatherese; (iv) however, salivary cortisol increased after still face confirming the stressful contribution of the experiment.

Computational methods can also be suitable for studying emotional communication [133]. Messinger [134] applied machine-learning to face-to-face interaction to explore the predictability of infant and mother smiles. The results measuring facial action [52] showed that (1) infant and mother smile activity exhibited changing (non-stationary) local patterns of association, suggesting the dyadic repair and dissolution of states of affective synchrony and (2) the duration of gazes at and away from the mother's face were positively predicted by the durations of the two previous gazes [135]. Together, results revealed that infants exhibit distinct and temporally stable levels of interest in social and non-social features of the environment.

In the context of an ongoing treatment study for neglected mothers (http://synedpsy.isir.upmc.fr/), we propose to study early interactions in synchronous and dyssynchronous dyads with a similar multidisciplinary approach using social signal processing. The parent and child will be video-recorded during free-play at baseline, after 6 months of treatment and at 1 year follow-up. The video recording of the free-play interaction will be coded with several computational tools able to measure motherese [129], speech turn-taking [132], joint attention [136] and movement coordination through skeleton extraction from a RGB-D sensor (Kinect) [137]. In parallel, clinical assessment tools including the CIB will be utilized to provide a global and valid assessment of synchrony. Components such as 0–3 diagnosis, social support or maternal insightfulness will also be assessed.

## Conclusions

Synchrony is a key feature of mother-infant interactions. It is not simple to objectively examine synchrony with currently available assessment tools due to its temporality and multimodal expression. However, irrespective of which assessment methods are used, it appears that synchrony should be regarded as a social signal *per se*, as it has been shown to be valid in both normal and pathological populations. Better mother-child synchrony is associated with familiarity (vs. unknown partner), a healthy mother (vs. pathological mother), typical development (vs. psychopathological development), and more positive cognitive and behavioral outcomes among children. Because mother-infant interactions are not static, an interactional model for the measurement of synchrony will need to capture the dynamic nature of the relationship and the flow of the interaction over time. We propose an integrative approach combining clinical observation and engineering techniques (e.g., social signal processing) to improve the quality of synchrony analysis.

## Supporting Information

Checklist S1 **PRISMA Checklist.**
(DOC)
